# Health technology assessment and value: the cancer value label (CAVALA) methodology

**DOI:** 10.3332/ecancer.2016.685

**Published:** 2016-10-28

**Authors:** Francisco Rocha-Gonçalves, Marina Borges, Patrícia Redondo, José Laranja-Pontes

**Affiliations:** Portuguese Institute of Oncology – Porto, Porto 4200-072, Portugal

**Keywords:** cancer, health technology assessment, economic evaluation, quality-adjusted life years, health care costs, outcomes measures

## Abstract

In modern health care systems, the soaring prices of drugs pose at least three major challenges: the growing economic burden of diseases, the uncertainty regarding innovation in health care, and the use of generic drugs and new indications.

In this context, the assessment of health care technology is not just about drugs, it is about ensuring that the system’s resources, namely financial, yield maximum health benefits. So, the assessment is about relating inputs with outputs; and also, resources with health-related outcomes. However, this method is based on specific assumptions and has its shortcomings.

This paper proposes a methodology called Cancer Value Label (CAVALA) which is a holistic and flexible concept of value. CAVALA overcomes the rationale that suffers from the communicational trap of having to discuss money versus life years gained. Some examples of CAVALA demonstrate that it has the potential to support health care decisions.

Using a step-by-step approach, we show how CAVALA can be implemented and further extended. We discuss its main uses to assess outcome selections, the pricing of drugs, and the decisions on the reimbursement of new drugs and indications.

## Introduction

In health care, the needs are growing and the resources are increasingly limited. Generally, technological innovations involve cost increases. An economic evaluation of innovations is crucial in order to make informed decisions and to maximise their value for society. This type of evaluation consists in comparing alternatives in terms of costs and effects [1]. At this point, the question is: what are the practical applications of these innovations, their implications, and their value as different therapeutic options in oncology?

This debate needs to continue in order to provide better tools for performing a health technology assessment (HTA). In fact, the focus on resource allocation has caused HTA – particularly, cost-effectiveness studies and the incremental cost-effectiveness ratio (ICER). The purpose is to provide evidence on the relationship between money and the life years saved. But the focus on HTA can threaten a rational, coherent, and thoughtful debate over the relative merits of novel health technologies.

So, the problem that this paper highlights is: how to enhance this focus over a larger range of factors that are key to the assessment of technology?

## Value in health care

Porter [2] finds that ‘Value’ is a concept that should be defined around the patient. As a result, he adds that, ‘in a well-functioning health care system, the creation of value for patients should determine the rewards for all other actors in the system’. It becomes clear that value in healthcare is measured by the outcomes achieved – outcomes that actually matter to the patient – not the volume of services delivered. Value is also defined as the outcomes relative to costs as in Equation 1.

This contribution from Porter is important because it shifts the focus from volume to value. This shift is a challenge both in the United States and Europe, because hospitals’ IT management does not have the tools to comply completely with the huge task of collection, integrating, and analysing this shift entails. Equation 1 shows that value is created through the process of care by investing resources that should be, in general, generating a significant improvement in patient-relevant clinical outcomes. That equation can be both an actual indicator and a conceptual representation of what direction to take in healthcare delivery: boosting value for patients out of invested resources.

But if one hopes to measure the performance of the care process by using selected outcomes, one should look into variables such as clinical outcomes (not surrogates, but real targets of therapeutic strategies at the patient level) and patient reported outcomes. This is important because the patient, despite the asymmetry of information that the health economics literature has long recognised, is the beneficiary of the investment. For example, if a new treatment causes different side effects, the patient needs to be asked about his or her experience and how he or she values (or not) the efforts in providing that therapeutic strategy. Patients’ and clinicians’ do not report disutility in the same way [3]. So, the better perspective of the patient, together with clinical evidence of the results, is critical to obtain a perspective on a technology. Equation 2 proposes a means to this objective by using a validated quality of life scale for the purpose of capturing patient reported outcomes.

Equation 1. Value defined as outcomes relative to costs.$\mathrm{Value}\;=\;\frac{\mathrm{Health Outcomes (Life years; QoL; etc)}}{\mathrm{Resources (costs) for delivering those outcomes}}$

Equation 2. Value measured using selected outcomes.$\mathrm{Value}\;=\;\frac{\mathrm{Clinical results}\;\times \;\mathrm{QoL}}{\mathrm{Resources invested}}$

The term ‘costs’, like in Porter, should include more than just the direct costs of a treatment. In fact, some savings may occur in a specific area of care if appropriate spending is done in other areas (e.g. investing in diagnosis technology and tests, so as to prevent late-stage cancers). Likewise, ‘costs’ also indicate the management of side effects – they are a contingency of the treatment, so their cost is a non-desired consequence. These cost should be taken into account both from the perspective of money and from the patients’ validation through a quality of life questionnaire. They can also include indirect costs; for example, if the methodology or objectives demand that the value of care is fully acknowledged.

## On HTA and its merits and limitations

The European Network for Health Technology Assessment (EUnetHTA) defines HTA as ‘a multidisciplinary process that summarises information about the medical, social, economic, and ethical issues related to the use of a health technology in a systematic, transparent, unbiased, robust manner’ [4].

It has a number of uses, including being an integrated part of the decision-making process that sets the price or reimbursement of health technologies; an input into market access decisions; and establishing guidance on the appropriate use of products. As a result, the HTA is performed by a variety of countries, systems, organisations, and others, such as the NICE, the SMC, the INFARMED, or the IQWIG. Many users see some of the techniques as especially useful for prioritising decisions on resource allocation. However, the HTA can assess a wide range of factors; the most common techniques concentrate on combining clinical outcomes and their cost-effectiveness by using techniques like ICER.

Its operation involves the identification, measurement, valuation, and comparison of the costs and consequences of the alternatives under analysis [1]. There are a variety of approaches, such as a cost analysis, cost-effectiveness analysis, cost-utility analysis, and a cost-benefit analysis, that are comparable in terms of cost but differ in how the consequences are measured and valued [1]. In the cost-effectiveness analysis, the consequences are measured in ‘natural units’, where it is necessary to consider the following points when selecting the indicators: the objectives of the interventions [5], the objectives of those who will actually decide [1], and the health gains for patients [5].

In the cost-utility analysis, the consequences or health outcomes are commonly valued in healthy years, such as quality-adjusted life-years (QALYs). The QALYs act as qualitative indicators that combine the quantity (mortality) and quality of life (morbidity) [1].

Further, in the cost-benefit analysis, the consequences are measured in monetary units.

With regard to costs, the most common classification considers the following types: direct costs (medical and non-medical), indirect costs or productivity losses (mortality and morbidity), and intangible costs [1, 6–7]. The costs to consider in the analysis depend on the perspective, for example, the health care provider’s, Ministry of Health’s, the State’s, or society’s [1].

Occasionally, when comparing the costs and consequences of new technologies (namely medicines), a new technology might be more effective and less expensive than the existing one. The decision maker has to decide whether to adopt the new treatment. The most used instrument, the ICER, is based on a trade-off between costs and effectiveness which is calculated in Equation 3 [5].

To study the economic assessments of drugs, the methodological guidelines of INFARMED provide several types of evaluations [8]. If the relevant consequences that are associated with different alternatives are the same in a study, then a minimisation of cost analysis can be used [8]. Otherwise a cost-effectiveness analysis should be used [8]. However, where practicable, a cost-utility or cost-benefit analysis is advisable in order to compare the results from pathology studies, with preference for the first [8].

Equation 3. ICER.$$\begin{array}{c} \mathrm{ICER}\;=\;\frac{\mathrm{Ca}-\mathrm{Cb}}{\mathrm{Ea}-\mathrm{Eb}}=\frac{\Delta C}{\Delta E}\mathrm{, where}\\ Ci-\mathrm{intervention cost i;}\\ Ei-\mathrm{intervention effectiveness i.} \end{array}$$

However, these approaches have been criticised for not being transparent or consistent. Hence, in the United Kingdom, HTA systems have proposed the implementation of value-based pricing (VBP) for health technologies that formally incorporate considerations for the disease’s severity and the innovativeness of the technology, its social benefits as well as cost-effectiveness to address these concerns. However, the proposal has not been acted on yet.

The existence of a public institution that carries out the economic evaluation of new therapies instead of a National Health Service is common in many countries. Schnipper *et al*. [9] summarise this process for the United Kingdom, Canada, Australia, France, and Germany. They report that the metrics that are most frequently used to evaluate the value of medical therapies are the QALYs and ICER. However, both have limitations.

One of the problems associated with the ICER is the definition of an acceptable maximum value at which the therapy is not cost–effective [9]. Currently, no uniform value exists among countries, and the countries that have adopted a value face questions on the limiting of patient choice and the rationalisation of health care [9]. Additionally, ICER is a ratio and a relative value can be misleading as to the absolute value of actual ‘life gains’ or ‘cost impact’. This is probably the reason why ICER should only be used as an aid to set a hierarchy among possible choices – but even in this role it does not perform adequately, since the options are not available when the regulators make their decisions.

The use of QALYs, which measure the patient’s individual decision process, have significant limitations because individuals with the same disease may have different preferences regarding their health [9].

McPake and Norman [10] state that two practical problems exist in an economic evaluation – measuring the costs and consequences. We add a third which is the comparison of various alternatives.

Recently, ASCO (American Society of Clinical Oncology) proposed a new ‘framework’ to assess the value of different therapeutic options in oncology that uses, among others, the following arguments [9]: not only is the cost of cancer treatment increasing but it has one of the highest growth rates compared to other diseases; in some cases, the adoption of newer and more expensive diagnostic and therapeutic interventions cannot be properly supported by medical evidence, thus increasing costs without improving the results; there are studies that show that patients want to receive financial information about the different treatment options, together with information on effectiveness and toxicity; patients tend to overestimate the benefits of a treatment, which sometimes increases overall survival by only weeks or months, or may even have no impact; and there are doubts among oncologists about whether and how treatment costs should affect their recommendations.

The reasons that led ASCO to propose a new ‘framework’ make the existence of research in this area an urgent matter in Europe. In this process, some questions have arisen, such as how to incorporate variables – for example, whether the disease is terminal or not; treatment access; varying population sizes; several drug indications; whether an intervention is curative, palliative, adjuvant, or preventative; budgetary constraints; overall economic impact; evidence of quality of life; and other factors such as politics. This article addresses some of these issues by delivering a framework that involves concepts that are currently under use, and expands the way they are used in HTA.

In the absence of tools for measuring the magnitude of the clinical benefit of anticancer drugs, ESMO (European Society for Medical Oncology) developed a Clinical Benefit Scale (ESMO-MCBS). This tool reliably develops a ranking of the magnitudes of clinically significant benefits that can be predictable from a new anti-cancer treatment [11]. With this instrument, ESMO takes ‘an important first step to the critical public policy issue of value in cancer care, helping to frame the appropriate use of limited public and personal resources to deliver cost-effective and affordable cancer care’ [11].

Both ESMO and ASCO have developed significant work to lead the health care community in measuring the value of oncology treatments by proposing their value assessment frameworks. Nevertheless, the frameworks are not equal and there are numerous examples where treatments seem to be valued differently [12].

However, there are other international groups developing equally important work related to value frameworks applied to oncology products.

The ICER group has developed a basic framework for assessing value that allows the classification and integration of various concepts in two different approaches: ‘care value’ and ‘provisional health system value’. The ‘care value’ approach focuses on four basics: comparative clinical effectiveness, incremental costs for results achieved, other benefits or disadvantages, and contextual considerations. This approach highlights the individual and his or her benefits. The ‘provisional value health system’ approach is based on assumption care value that integrates the potential impact of a new intervention in the short-term budget. Within ICER, the theoretical threshold for the budget impact is based on the willingness of society to pay [13].

## HTA: IPO Porto approach – the CAVALA model

In a step-by-step approach, this section illustrates CAVALA’s rationale, implementation, and decision-support role.

## Objectives and method

Currently, IPO Porto is developing a methodology for drug assessment and the reporting of results, which it has extensive experience in. Some practical applications of this methodology highlight that it can have a significant role in supporting decisions relating to health care.

While this methodology assesses the degree of innovativeness in technologies, it also mitigates the main limitations associated with the methods currently used in Portugal. Although it can expand into other disease areas, the methodology is named CAVALA – Cancer Value Label.

CAVALA starts with the assumption that the value of a treatment is measured by the relationship between its results and the amount of resources it requires [2]. So the question is: what do we want for patients from the perspective of a sensitive provider? The answer is: more years of life and a higher quality of life. On the other hand, we do not want the costs to be disproportionate to the benefits of the treatments.

**Figure 1.**
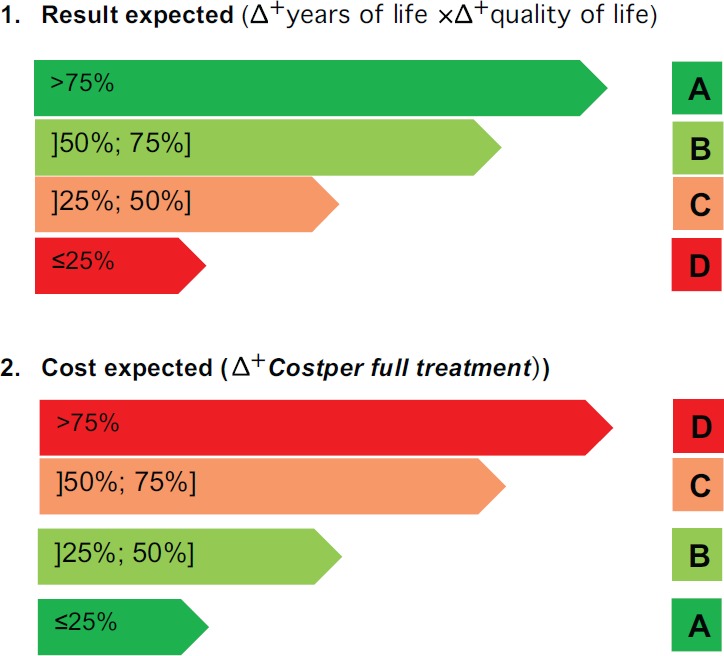
Costs and outcomesgrades.

To operationalise this concept of value, we propose the decision grid shown in Figure 1. The grid compares a candidate technology and an incumbent or another comparator by analysing two parameters:

- What are the expected outcomes?
- What are the expected costs?

Displaying the two parameters in the following diagram shows that drug Y only has results for 20% or less patients, but its costs compared to a similar medication are 10 times higher:

**Figure:**
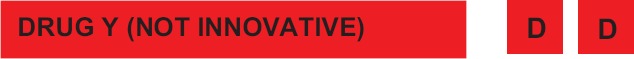

**Figure 2.**
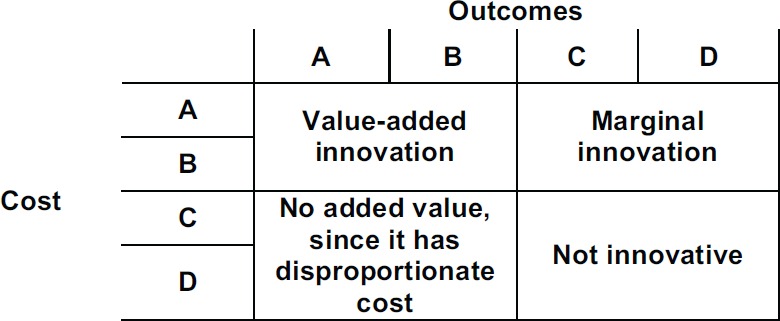
Costs and outcomes matrix.

According to CAVALA, both medications can be clearly innovative and value-added treatments that combine classification A or B simultaneously from the perspective of costs and outcomes. Combining the parameter outcomes and costs produces the grid shown in Figure 2.

If x is a drug with the following outcomes,

Outcomes: over 38% → C

Cost of which: 380% → D, then the following result is obtained:

**Figure:**
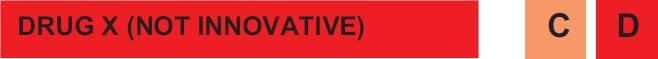

These scales are not just to rank options. They also clearly communicate the difficulties in recognising the innovative nature of new products for a wider community of stakeholders at different levels of technical knowledge.

In the example above (drug X), the price is disproportionate to the benefits (+ 380% vs. + 38%) that prohibits a better classification of the product and its eligibility for the purposes of hospital and drugstore formularies.

The proposed model is for the evaluation of new technologies. If the ‘entrant’ is a generic drug, then a cost minimisation analysis could be used. The great asset of the CAVALA is that it allows the addition of other variables, such as overall survival (OS), progression-free survival (PFS), and duration of treatment. For treatments that cover multiple cancers, other outcome measures, like DALYs, can always be used. But we have tried to make the model as stepwise as possible.

## Examples

As described earlier, the intention is to analyse all relevant costs to help the payer to make decisions. The aim is to start with direct costs but to also address the costs from side effects, such as the patient’s productivity. The indirect costs, if available and/or relevant to the analysis, can be added.

By using published data from two evidence review reports that are available at the National Institute for Health and Care Excellence’s (NICE) website, we can demonstrate in practice the methodology. For the purpose of this illustration, we consider the drug bendamustine as a valid substitute for chlorambucil.

As explained before, in order to apply the methodology, we combine the differential impact on the quality-adjusted life years with the differential cost per treatment.

**Figure 3.**
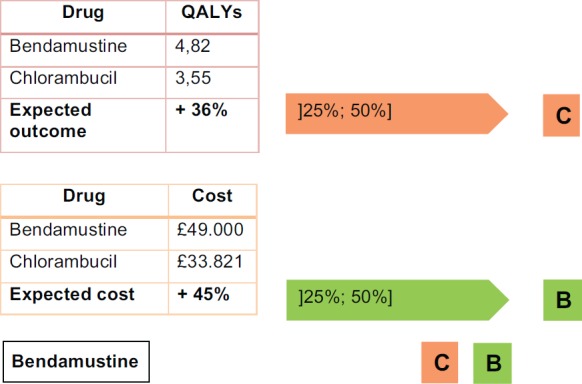
Practical example of IPO Porto methodology – bendamustine.

According to the report presented by Hoyle *et al*. [14], patients that take bendamustine as a first-line treatment instead of chlorambucil for chronic lymphocytic leukaemia (Binet stage B or C) have the results shown in Figure 3.

**Figure 4.**
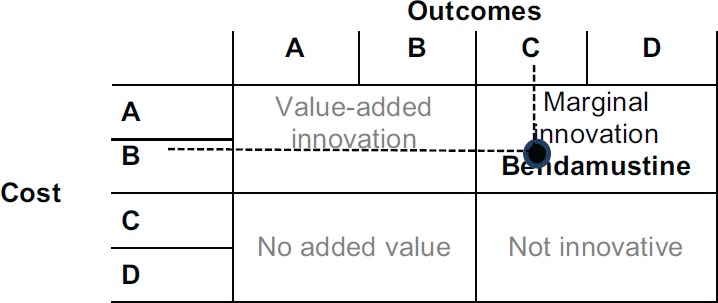
Bendamustine costs and outcomes matrix.

Combining the parameter outcomes and costs, bendamustine is a marginally innovative medicine. Figure 4 shows the result.

The NICE recommends bendamustine as a possible treatment for some people with chronic lymphocytic leukaemia in Binet stage B or C.

This recommendation is fully compatible with the CAVALA methodology because there is a clear improvement in the QALYs (+36%, close to one QALY). The increase in the costs of the full treatment are commensurate with this benefit. This conclusion shows the validity of the IPO Porto methodology.

**Figure 5.**
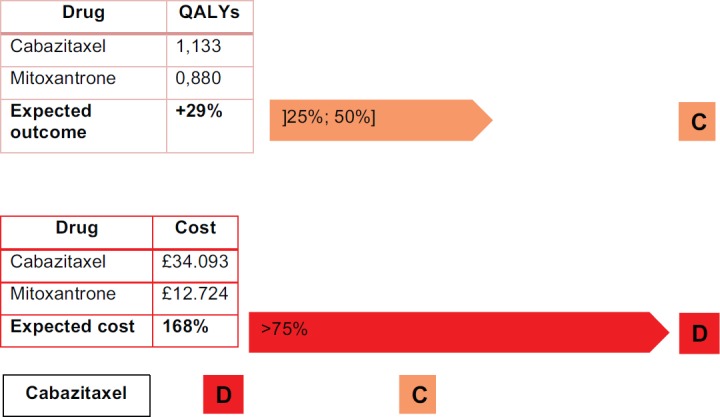
Practical example of IPO Porto methodology – cabazitaxel.

In another example, we can test this methodology using a report by Stevenson *et al*. [15] who review the effectiveness of the medicine cabazitaxel compared to mitoxantron for the second-line treatment of hormone refractory, metastatic prostate cancer. The results are shown in Figure 5.

**Figure 6.**
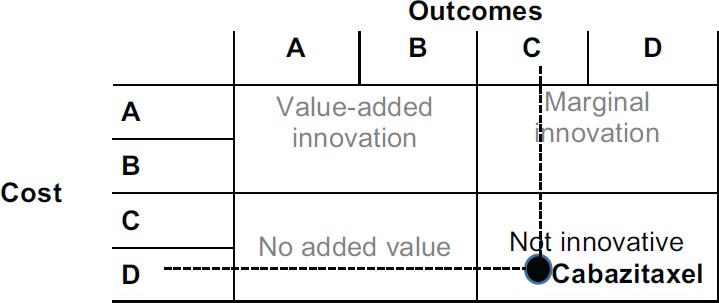
Cabazitaxel costs and outcomes matrix.

Cabazitaxel is not an innovative drug, as Figure 6 shows. It has a simultaneously high cost per treatment and a small impact in terms of QALYs (the selected outcome).

With cabazitaxel, in order to have a 29% increase in QALYs requires doubling the costs to the patients.

In order to fall into an ‘acceptance region’ for the IPO Porto methodology, cabazitaxel should have a cost very close to, or even less than, the comparator. Otherwise, a decisive argument does not exist because of the low evidence of effectiveness and quality of life.

## Adding new dimensions of the analysis

The proposed framework, Equation 1, does not need to be very rigid. In fact, the description of the outcomes and the resources is disease-specific or even system-specific, especially at the international level. For example, in Kaplan *et al*. [16], a set of different spider-web graphics are used to depict several outcomes.

Costs are one axis of the graphic with patient reported outcomes (quality of life subdimensions like urinary or sexual symptoms of a treatment in a urology context) and the clinical outcomes (surgical and radiation complications; survival and diseases recurrence) are the others.

**Figure 7.**
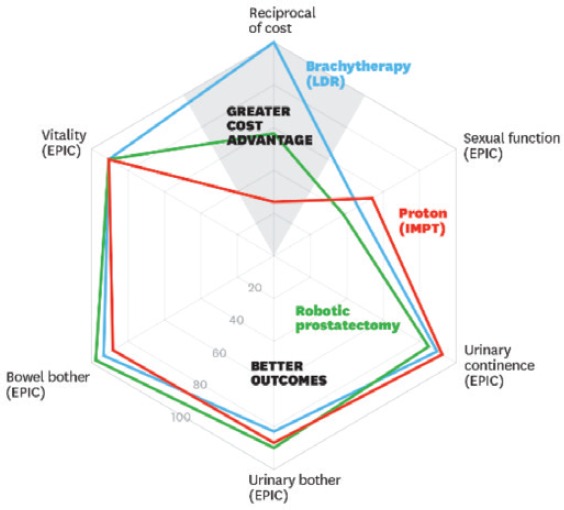
Comparing the value of three alternative prostate cancer treatments [13].

The CAVALA methodology is also compatible with more variables. However, it becomes impossible to represent it graphically as in Figure 7 because it requires more than two dimensions. But we can make an analogy: as long as the treatment does not go under 50% on each axis variable, it remains a valuable alternative from the perspective of outcomes and resources. In order to show added value, the area within the ‘spider web’ most be as large as possible.

## Discussion: decision making with the CAVALA

Practically, CAVALA should be able to support decisions on pricing (as explained in subsection 4.1. with drug x) and on setting criteria for including drugs in hospital formularies. Health technologies, including drugs, are clinically effective if they provide a general health benefit, despite the adverse events, when compared with alternative treatments.

The CAVALA model should support decision-makers in drawing assumptions from a set of facts, independently of each decision process algorithm. With CAVALA, each decision-maker should select the information items that he or she values in the assessment, like the examples described earlier.

So, decision-makers have to make decisions when determining whether technologies are effective clinically and costwise. It is crucial that the method used as an instrument is not an instruction. Recommendations must be established on scientific findings about the effectiveness both clinically and costwise, but also to take into account social preferences as expressed by social value conclusions.

## Conclusions

Clearly, value in health care needs to be discussed from a holistic perspective. The effects of treatments, as well as the burden of diseases, affect the economic and social lives of patients. As a result, one should be able to capture value fluctuations in any of these areas.

We have combined a discussion on value with one on the HTA. This is important because both analyses make sense: if we are to evaluate and compare the relative or added value of technologies, is value as measured in Equation 2 the key attribute to measure appraisals?

We were able to escape a common conceptual and communicational trap, which is the apparent trade-off between money and the time of life for patients. We are able to do so because of the thresholds discussion and the rigidity of the cost-effectiveness analysis. The ICER-based analysisis currently being used as a means to lower the pricing of new drugs – given that we have no better tools to value them. But CAVALA offers an improved rationale. According to Figure 2, we still acknowledge the full clinical value of a drug that is deemed as nonvaluable because of an excessive price tag. However, it becomes clear that the promoter of the drug should bring the price down to a proportionate ratio commensurate with the benefits to the patient’s condition.

We discussed value and carried the discussion to the point where it becomes relevant to patients and providers. This is possibly a good step to bring them closer to the HTA process, improving participation – for example on deciding which outcomes to measure – and in turn making economic analysis of health technologies a well-accepted, mainstream function within drug development.

## Conflict of Interest

The authors state that there are no financial, labour, or other relations that pose a conflict of interest regarding this study. That is, we have not received any ‘benefits in property, hospitality, or subsidies’.
